# Investigating the drivers of the spatio-temporal heterogeneity in COVID-19 hospital incidence—Belgium as a study case

**DOI:** 10.1186/s12942-021-00281-1

**Published:** 2021-06-14

**Authors:** Simon Dellicour, Catherine Linard, Nina Van Goethem, Daniele Da Re, Jean Artois, Jérémie Bihin, Pierre Schaus, François Massonnet, Herman Van Oyen, Sophie O. Vanwambeke, Niko Speybroeck, Marius Gilbert

**Affiliations:** 1 Spatial Epidemiology Lab (SpELL), Université Libre de Bruxelles, 50 av. FD Roosevelt, 1050, CP160/12 Bruxelles, Belgium; 2 Department of Microbiology, Immunology and Transplantation, Laboratory for Clinical and Epidemiological Virology, Rega Institute, KU Leuven - University of Leuven, Leuven, Belgium; 3 Institute of Life-Earth-Environment (ILEE), Université de Namur, Rue de Bruxelles 61, 5000 Namur, Belgium; 4 NAmur Research Institute for LIfe Sciences (NARILIS), Université de Namur, Rue de Bruxelles 61, 5000 Namur, Belgium; 5 Department of Epidemiology and Public Health, Sciensano, Brussels, Belgium; 6 Earth & Life Institute, Georges Lemaître Centre for Earth and Climate Research, UCLouvain, Place Louis Pasteur 3, 1348 Louvain-la-Neuve, Belgium; 7 ICTEAM, UCLouvain, 1348 Louvain-la-Neuve, Belgium; 8 Public Health and Primary Care, Gent University, Gent, Belgium; 9 Institute of Health and Society (IRSS), Université Catholique de Louvain, Clos Chapelle-aux-champs 30, 1200 Brussels, Belgium

**Keywords:** COVID-19, Hospitalisation incidence, Spatial covariates, Temporal covariates, Boosted regression trees, Belgium

## Abstract

**Background:**

The COVID-19 pandemic is affecting nations globally, but with an impact exhibiting significant spatial and temporal variation at the sub-national level. Identifying and disentangling the drivers of resulting hospitalisation incidence at the local scale is key to predict, mitigate and manage epidemic surges, but also to develop targeted measures. However, this type of analysis is often not possible because of the lack of spatially-explicit health data and spatial uncertainties associated with infection.

**Methods:**

To overcome these limitations, we propose an analytical framework to investigate potential drivers of the spatio–temporal heterogeneity in COVID-19 hospitalisation incidence when data are only available at the hospital level. Specifically, the approach is based on the delimitation of hospital catchment areas, which allows analysing associations between hospitalisation incidence and spatial or temporal covariates. We illustrate and apply our analytical framework to Belgium, a country heavily impacted by two COVID-19 epidemic waves in 2020, both in terms of mortality and hospitalisation incidence.

**Results:**

Our spatial analyses reveal an association between the hospitalisation incidence and the local density of nursing home residents, which confirms the important impact of COVID-19 in elderly communities of Belgium. Our temporal analyses further indicate a pronounced seasonality in hospitalisation incidence associated with the seasonality of weather variables. Taking advantage of these associations, we discuss the feasibility of predictive models based on machine learning to predict future hospitalisation incidence.

**Conclusion:**

Our reproducible analytical workflow allows performing spatially-explicit analyses of data aggregated at the hospital level and can be used to explore potential drivers and dynamic of COVID-19 hospitalisation incidence at regional or national scales.

**Supplementary Information:**

The online version contains supplementary material available at 10.1186/s12942-021-00281-1.

## Background

COVID-19 (coronavirus disease 2019), caused by a new coronavirus (severe acute respiratory syndrome coronavirus 2; SARS-CoV-2), was first reported in early December 2019 in the province of Hubei in China [1] and followed by a fast and extensive spread throughout the world [2]. On March 12, 2020, the World Health Organisation (WHO) declared COVID-19 a pandemic. The COVID-19 pandemic has resulted in considerable public health, social and economic disruptions [3, 4]. Moreover, the pandemic has threatened the saturation or actually led to the saturation of national hospitalisation systems. In order to mitigate and manage epidemic surges, it is of strategic importance to disentangle the spatio-temporal dynamic and potential drivers of COVID-19 hospitalisation incidence. In this context, one practical limitation can be the lack of access to spatially-explicit health data like, for instance, the geographic origin of hospitalised patients. To circumvent this issue, we here propose an analytical framework based on the definition of hospital catchment areas (HCAs), a concept that has previously been used to study the geographic access to healthcares in the context of the COVID-19 epidemic in Brazil [5]. The overall objective of our study is to present and describe an analytical framework based on the delimitation of such HCA units and that allows to analyse the dynamic of potential drivers of COVID-19 hospitalisation incidence in a spatially-explicit context. As a study case, we illustrate our approach on the study of the COVID-19 epidemic during the year 2020 in Belgium, for which we precisely had access to hospitalisation incidence data at the hospital level.

In Belgium, the first COVID-19 case was officially reported on February 4, 2020, in an asymptomatic Belgian citizen repatriated from Wuhan [6]. Symptomatic cases increased from March onwards, initially related to the return of holidaymakers, mainly from Italy [7]. This was soon followed by local transmission and extensive spread in the country [8]. The Federal Government implemented social-distancing measures and eventually a full lockdown during March–May 2020, which led to the end of the first wave. The summer months showed a lower incidence of positive cases and hospitalisations, in Belgium as in other European countries. However, September 2020 saw a slow but steady growth of the registered cases and new hospitalisations, which resulted in the start of the second wave at the end of September and brought the Federal Government to tighten up the social distancing measures at the end of October. With now more than 20,000 confirmed COVID-19 deaths, Belgium has been highly impacted by the two epidemic waves occurring during spring and fall 2020, with a mortality rate among the highest in the world. While explanatory factors like population density, connectivity, age pyramid or the critical circulation of the virus in nursing homes [9] have been discussed, the exact causes of one the highest COVID-19 mortality in the world (172 deaths/10,000 people, https://coronavirus.jhu.edu/data/mortality) has still to be more thoroughly understood, which could require analytical comparisons between countries. As illustrated by the New York Times in an article [10] highlighting excess mortality likely due to underreporting COVID-19 deaths, such a comparison will however be complicated by differences among countries in terms of reporting of COVID-19 mortality. In Belgium, the COVID-19-induced mortality has been monitored relatively well, as reflected by the high correlation between excess mortality statistics for 2020 and the COVID-19 recorded deaths [11]. Besides a high mortality, with more than 50,000 hospital admissions, Belgium also experienced an important hospital incidence during the two first waves of the COVID-19 epidemic, with marked variations from one hospital to another. During the peak surge, the intensive care unit (ICU) thresholds were locally exceeded, causing the need to supplement ICU beds in emergency. On many occasions, these overflows adversely impacted the fate of critically-ill COVID-19 patients [12]. With several weeks of hindsight and the availability of quality-controlled data, we can now investigate the potential drivers of the spatial–temporal heterogeneity in COVID-19 hospitalisation incidence in Belgium.

In the present study, we specifically aim at (i) comparing the spatial patterns of hospitalisation incidence between the two epidemic waves; (ii) understanding if spatial variations in hospitalisation incidence is related to population, economic, or environmental covariates (population density, number of nearby nursing home residents, median age, percentage of workers working in the primary/secondary/tertiary sectors, median declared income, ratio of urban areas, local concentration of fine particles); and (iii) exploring if temporal variations in hospitalisation incidence might be related to some temporal covariates such as human mobility, climatic factors (temperature, humidity, and solar radiation), or environmental factor (air quality).

## Methods

### Definition and delimitation of hospital catchment areas

The Belgian national public health research institute (Sciensano) collected and aggregated COVID-19 hospitalisation data according to an established hospital surveillance protocol [13]. Although some data are available at the municipality level, other important data sets were only collected per hospital (*n* = 103). This forced us to work at the level of HCAs. The hypothesis underlying the concept of HCA is that patients normally choose the hospital closest to their home. While the simplest approximation of spatial accessibility is Euclidean distance, a more realistic way to generate accessibility maps is to estimate travel times between populations and hospitals. In practice, we used the friction surface developed by Weiss et al. [14] to derive maps of travel time between any 1 km^2^ pixel in Belgium and each of the 103 hospitals. Each pixel was assigned the hospital that minimised such travel time, and the set of pixels attributed to a given hospital was defined as its catchment area. In areas where multiple hospitals could be found within the same pixel of the friction surface, catchment areas and associated epidemiological data were aggregated (*n* = 2). The resulting HCAs were used as working units in all subsequent analyses based on three different time periods: a time period corresponding to the first (01/03–31/05/2020) and second (01/09–30/11/2020) epidemic waves, as well as a time period covering the entire Belgian epidemic until the end of November 2020 (01/03–30/11/2020). Specifically, we worked with a measure of hospitalisation incidence (HI) computed as the cumulative number of new hospitalisations per 100,000 inhabitants for each HCA and time period.

### Investigating the drivers of the spatial heterogeneity in COVID-19 hospitalisation incidence

We investigated the extent to which the HI heterogeneity among HCAs could be explained by spatial covariates using three different categories of analyses: (i) visual explorations based on principal component analyses (PCAs), (ii) univariate correlation analyses summarised with a correlogram and estimated through univariate linear regression (ULR) analyses, and (iii) multivariate analyses conducted with multivariate linear regression (MLR) and machine learning approaches. The machine learning approach consisted in using a boosted regression tree (BRT) algorithm [15] allowing the estimation of non-linear relationships between response (HI) and predictive variables (spatial covariates) by generating a collection of sequentially fitted regression trees to optimise the predictions. The PCA was performed with the dudi.pca function of the ade4 R package, and the correlogram visualisation was generated with the heatmap.2 function of the R package gplots. We also performed spatial autocorrelation tests using the Moran.I function of the R package ape. Regarding BRT analyses, they were carried out using the R package dismo. To select the optimal number of trees in the BRT models, we used a cross-validation procedure based on 5 separated folds. All BRT analyses were run and averaged over 10 cross-validated replicates, with a tree complexity set at 5, an initial number of trees set at 10, a learning rate of 0.005, and a step size of 5.

**Fig. 1:**
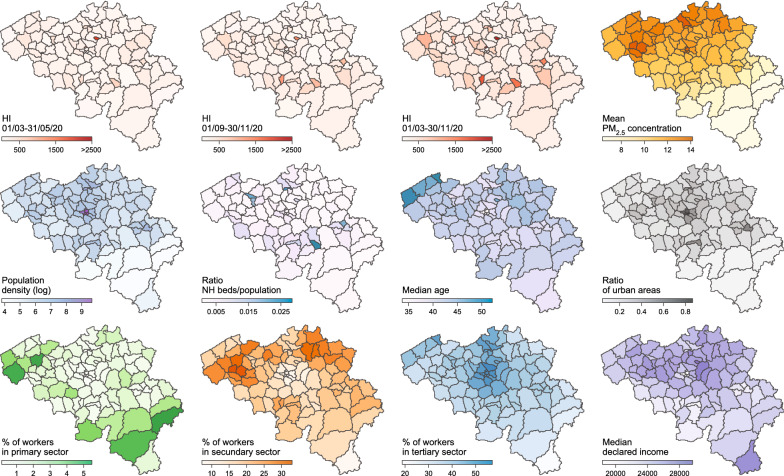
Spatial covariates tested as potential predictors of the heterogeneity in hospitalisation incidence. PM_2.5_ refers to particulate matter of ≤ 2.5 μm. Median declared income is expressed in euros (€) and was averaged over each HCA (see the Methods section in Additional file for further detail). Mean concentration of particulate matter of ≤ 10 μm (PM_10_) and proportion of people who are more than 65 years old are not represented but are highly correlated with mean PM_2.5_ concentration and median age, respectively

We analysed eleven distinct spatial covariates as drivers of the spatial heterogeneity of HI (Fig. 1): human population density computed from the human population raster made available by the WorldPop project (https://www.worldpop.org), the median age and the proportion of people older than 65 years, the median income, the percentages of workers in the primary, secondary or tertiary sector, all provided by the Belgian Federal Public Service Economy (SPF Economie), the ratio nursing home (NH) beds/population computed from the number of NH beds provided at municipal level by the Belgian Federal social care agencies (https://www.zorg-en-gezondheid.be for Flanders, http://sante.wallonie.be for Wallonia, and http://www.iriscare.brussels for Brussels), the concentration of particulate matters ≤ 2.5 or 10 μm (PM_2.5_ and PM_10_) provided by the Belgian Interregional Environment Agency, and the proportion of urban areas computed from the European land use raster provided by the Corine Land Cover database (https://land.copernicus.eu). Median age, proportion of people > 65 years old, median income, percentage of workers in the primary, secondary, or tertiary sectors, and the number of NH beds were initially provided at the municipality level. We transposed these values to the HCA level by weighting the contribution of each municipality according to the number of people living in areas overlapped by both a given municipality and given HCA polygon. In practice, the value *v*_*HCA*_ assigned to a given HCA was computed as follows:

1 $${v_{HCA}} = \sum\limits_i {\left( {{v_{municipality,i}} \times \left( {\frac{{{p_{overlap,i}}}}{{{p_{HCA}}}}} \right)} \right)}$$

where *v*_*municipality,i*_ is the covariate value available for the municipality *i*, *p*_*overlap,i*_ is the number of people living in the area overlapped by the HCA and municipality *i*, and *p*_*HCA*_ is the total number of people living in the HCA under consideration. Estimates of PM_2.5_ and PM_10_ concentrations were provided in the form of geo-referenced grids averaged over the entire year 2017. We used the function exact_extract from the R package exactextractr to extract and assign PM concentration values to assign to each HCA.

### Investigating the drivers of the temporal variability in COVID-19 hospitalisation incidence

We subsequently investigated the extent to which HCA specific daily hospitalisation ratios (DHRs) could be related to temporal covariates. DHR values were computed on a time window of 7 days. For a given HCA, a DHR was defined by the ratio of total number of persons hospitalised at day *d* divided by the total number of persons hospitalised at day *d*-7, and are thus by essence temporally autocorrelated (Additional file 1: Fig. S1). We analysed five distinct temporal covariates (Additional file 1: Fig. S1): an index of human mobility, PM_2.5_ concentration, and three climatic variables (temperature, relative humidity, and solar radiation). Index values of human mobility were based on anonymised mobile phone data provided by Proximus, the largest mobile telecommunications company in Belgium. Proximus provided a mobility index per day and municipality computed as the ratio between the number of trips “in” or “out” the municipality, divided by the number of subscriptions for the municipality. The number of trips “in” and “out” were here defined as the number of journeys inside and outside the considered municipality, respectively. Specifically, the number of trips “in” measures the number of mobile phone SIM cards “living” in the considered municipality, and the number of trips “out” measures the number of mobile phone SIM cards not “living” in but “visiting” that municipality. The number of subscriptions is defined as the number of mobile phone SIM cards “living” in a municipality. Following data privacy protection, Proximus only provided a number of trips “in”/”out” when a sufficient number of at least 30 SIM cards were involved in a daily trip between two municipalities. The resulting index values of mobility were transposed from the municipality to the HCA level using the procedure described above. Estimates of PM_2.5_ concentration were obtained from the Belgian Interregional Environment Agency and provided in the form of daily geo-referenced grids. We again used the function exact_extract from the R package exactextractr to extract and assign PM_2.5_ concentration values to assign to each HCA.

Three atmospheric variables were considered to explain the observed variations in DHR. All three variables were retrieved from the ERA5 climate reanalysis [16]. A climate reanalysis consists of outputs from a numerical model of the atmosphere that has been constrained by in-situ measurements like sea-level pressure, near-surface air temperature and wind speed (among others), all obtained from in-situ measuring devices such as thermometers in weather stations or radiosondes operating on weather balloons. The prime advantage of using reanalyses is that they provide gridded information at high temporal frequency (up to hourly), which is not possible from the few weather stations available in Belgium alone. In the context of this study, for which regional scale near-real time estimates of atmospheric variables were required, the ERA5 reanalysis data (~ 35 km resolution) appeared as a better choice than the scattered point weather station data. The three variables retrieved from ERA5 are: (i) two-meter air temperature (units: K), (ii) the surface direct short-wave radiation flux (units: W m^−2^), (iii) the relative humidity at 1000 hPa, defined as the ratio between the water vapour pressure and the saturation water vapour pressure (in %).

## Results

### Comparing the hospitalisation incidence of the two epidemic waves

**Fig. 2:**
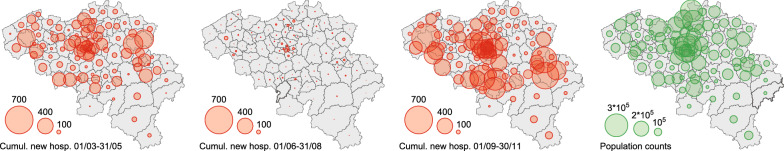
Spatial distribution of cumulative numbers of new hospitalisations per hospital catchment area (HCA). Cumulative numbers of new hospitalisations are here reported for three distinct periods: 01/03–31/05/2020 (corresponding to the first epidemic wave), 01/06–31/08/2020 (summer period), and 01/09–30/11/2020 (corresponding to the second epidemic wave). The rightmost panel maps the baseline population in each HCA

**Fig. 3:**
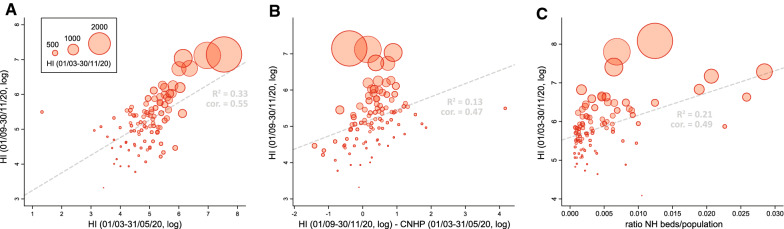
Exploration of the heterogeneity in hospitalisation incidence. The measure of hospitalisation incidence (HI) is computed as the cumulative number of new hospitalisations per 100,000 inhabitants for a given hospital catchment area (HCA) and a given time period. **A** Association between HI values of the second and first epidemic waves. **B** Association between HI values of the second epidemic wave and the difference between HI values of the second and first epidemic waves. **C** Association between HI values computed for the entire epidemic period (01/03–30/11/20) and the ratio between nursing home (NH) beds and population count in each HCA. Dot sizes are proportional to HI values computed for the entire epidemic period under consideration (01/03–30/11/20); R^2^ and “cor” refer to the coefficient of determination and the Spearman (rank) correlation

A first visual exploration of the absolute number of cumulated new hospitalisations per HCA and per time period confirmed that hospital admissions were globally higher during the second than the first epidemic wave and that, as expected, hospital admissions were lower in less populated areas of the country (Fig. 2). As a preliminary analysis, we performed Moran's I tests that did not lead to the detection of significant spatial autocorrelation for HI measures (for all time periods), meaning that there was no clear tendency for neighbouring HCAs to share similar hospitalisation incidence. Comparisons of HI values indicated that hospitalisation incidence was relatively well correlated between the two epidemic waves (Fig. 3A), but also that the hospitalisation incidence of the second wave tended to be correlated with the HI differences between the two epidemic waves (Fig. 3B). The latter results thus relate to a tendency to observe a higher hospitalisation incidence during the second wave in HCAs that were relatively less affected by the first epidemic wave.

### Investigating the drivers of the spatial heterogeneity in COVID-19 hospitalisation incidence

**Fig. 4:**
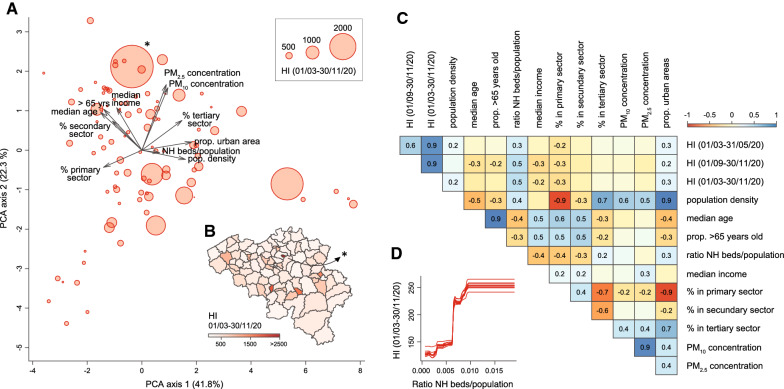
Analyses of the potential predictors of spatial heterogeneity in hospitalisation incidence (HI). **A** Principal component analysis (PCA) based on all spatial covariates, each dot corresponding to a distinct hospital catchment area (HCA; see also Additional file 2: Figure S2 for an alternative PCA that also includes HI variables). **B** Map of HCAs coloured by HI value computed for the entire epidemic period under consideration. **C** Correlogram reporting Spearman correlations among all spatial covariates and HI values for the three considered periods; only significant correlation values (*p*-values < 0.05) are reported. **D** Selected result from the boosted regression trees (BRT) analysis performed with all spatial covariates and HI values computed for the entire epidemic period as response variable: partial responses for HI values for the ratio of nursing home (NH) beds divided by the population in each HCA; i.e., the spatial covariate associated with the highest relative influence in the BRT model (~ 57%; Table 1). (*) indicates a potential HCA outlier discarded for the statistical analyses reported in Additional file 4: Table S2

Visual explorations based on PCAs (Fig. 4A, Additional file 2: Figure S2) did not yield a clear correlation pattern between HI and spatial predictors, even if we could observe that most HCAs with relatively higher HI values tended to be associated with higher local population density, proportion of urban areas, and ratio between NH beds and HCA population. In Fig. 4B, we could note that one of the HCA was associated with a particularly high HI value, which was likely due to the relatively small area defined around the hospital included in this area. As the resulting HCA could represent a potential outlier, we repeated the multivariate analyses after having excluded that specific HCA (see below). Overall, these PCA trends were further confirmed by the univariate analyses summarised in our correlogram (Fig. 4C): for all three periods considered (first and second epidemic waves, as well as the entire epidemic period until the end of November, 2020), HI values were positively correlated with the proportion of urban areas and the ratio NH beds/population, but also negatively correlated with the percentage of people working in the primary sector. In our correlogram, population density was, however, not significantly associated with HI during the second epidemic wave (Fig. 4C). In our ULR analyses, only two spatial covariates were associated with a significant coefficient of determination (R^2^) value for all time periods under consideration: the proportion of urban areas and the ratio NH beds/population (Table 1).

**Table 1:** Analyses of the potential predictors of spatial heterogeneity in hospitalisation incidence

	HI (01/03–31/05/2020)			HI (01/09–30/11/2020)			HI (01/03–30/11/2020)		
	MLR R^2^ = 0.18*, BRT cor. = 0.69			MLR R^2^ = 0.24*, BRT cor. = 0.76			MLR R^2^ = 0.23*, BRT cor. = 0.75		
Spatial covariate	ULR R^2^	MLR β	BRT RI	ULR R^2^	MLR β	BRT RI	ULR R^2^	MLR β	BRT RI
Population density	0.00	–	0.8%	0.00	–	1.1%	0.00	–	0.9%
Median age	0.00	–	2.6%	0.02	–	6.2%	0.00	–	2.4%
Prop. > 65 years old	0.00	–	6.7%	0.01	–	1.4%	0.00	–	1.3%
Ratio NH beds/population	0.15*	0.22*	48.1%	0.22*	0.41*	55.3%	0.21*	0.31*	56.8%
Median income	0.00	–	3.2%	0.05*	-0.07	5.5%	0.00	–	3.9%
% in primary sector	0.04	–	5.7%	0.04*	0.00	14.3%	0.05*	0.02	14.4%
% in secondary sector	0.02	–	2.2%	0.02	–	1.8%	0.00	–	2.4%
% in tertiary sector	0.05*	0.06	3.0%	0.01	–	2.5%	0.00	–	1.5%
PM_10_ concentration	0.01	–	1.6%	0.00	–	3.1%	0.00	–	1.7%
PM_2.5_ concentration	0.01	–	0.9%	0.00	–	1.8%	0.00	–	0.9%
Prop. urban areas	0.05*	0.05	25.4%	0.05*	0.09	7.1%	0.06*	0.12	13.7%

This table summarises the results of univariate linear regression (ULR), multivariate linear regression (MLR), and boosted regression trees (BRT) analyses performed to investigate the association between measures of hospitalisation incidence (HI) and various spatial covariates associated with hospital catchment areas (HCAs). We report the following metrics: the coefficient of determination (R^2^) for the ULR analyses, the regression coefficient (β) for the MLR analyses, and the relative influence (RI) associated with each spatial covariate for the BRT analyses. We also report the overall R^2^ and Spearman correlation (“cor.”) for each distinct MLR and BRT analysis, respectively. (*) indicates if a given R^2^ or β is significant (p-value < 0.05). See also Additional file 4: Table S1 for the results of the same analyses performed when only considering HI values based on nursing home (NH) residents, and Additional file 4: Table S2 for the results of the same analyses performed after having discarded HCAs of Brussels-Capital region as well as a potential outlier HCA (highlighted in Fig. 4)

To investigate the effects that the cross-covariate correlations may have on our results, we further performed multivariate analyses, which confirm the association between HI and the ratio NH beds/population. We first performed MLR analyses, which were only based on spatial covariates for which we got a significant R^2^ value from the corresponding ULR analysis. For the ratio NH beds/population, we estimated significant MLR coefficient (β) values ranging from 0.22 to 0.41 for the first and second epidemic wave, respectively (Table 1). On the contrary, we did not estimate significant MLR coefficients for the proportion of urban areas. It is however important to note that our MLR analyses only explained a relatively limited proportion of HI variability (R^2^ < 0.25; Table 1). Secondly, we performed BRT analyses to further explore non-linear relationships between HI and the different spatial covariates tested in this study. Our BRT models allowed reaching a relatively high correlation between observed and predicted HI values, with Spearman correlation values ranging between 0.69 and 0.76 for the first and second epidemic wave, respectively (Table 1). These BRT analyses mainly confirmed the notable contribution of the ratio NH beds/population in predicting HI values. Specifically, we got BRT relative influence (RI) values of 48 and 55% for the first and second epidemic wave, respectively, as well as of 57% for the entire epidemic period (Table 1). For the proportion of urban areas, RI values were much lower, except when considering the first epidemic wave for which we get a RI value of 25%. BRT response curves illustrate the positive yet not linear estimated relationship between HI and the ratio NH beds/population (Fig. 4D; and see also Additional file 3: Figure S3 for all response curves). We repeated all multivariate analyses only considering hospitalisations of NH residents (Additional file 4: Table S1) and excluding HCAs of Brussels-Capital Region and the potential outlier area highlighted in Fig. 4B (Additional file 4: Table S2). These two alternative sets of analyses confirmed the main results presented in Table 1.

In addition to exploring the association between HI and spatial covariates, we also used our BRT models to analyse the ability of those models to predict observed hospitalisation incidence. Specifically, we estimated the correlation between observed and predicted HI values, the latter being predicted under our different BRT models: the BRT model trained with HI values of the first epidemic wave, the BRT model trained with HI values of the second epidemic wave, the BRT model trained with HI values for the entire epidemic period under consideration, and a fourth BRT model trained with HI values of the second epidemic wave (01/09–30/11/2020) but also including HI values of the first epidemic wave as a potential predictor in addition to all the other considered spatial predictors (Additional file 4: Table S3). Our results indicate a good ability of this fourth BRT model to predict HI values for the second epidemic wave, which can in practice be useful for predicting upsurge in hospital incidence in case of further epidemic waves.

### Investigating the drivers of the temporal variability in COVID-19 hospitalisation incidence

**Fig. 5:**
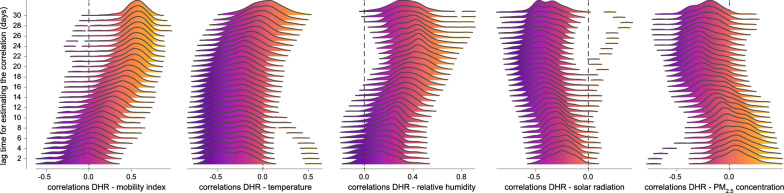
Investigating the drivers of the temporal variability in COVID-19 hospitalisation incidence. Each density plot reports the distribution of correlation values obtained when comparing daily hospitalisation ratios (DHRs) and a specific temporal covariate for the entire epidemic period under consideration (01/03–30/11/20). Each reported distribution gathers 103 correlation values, i.e. one correlation value (Spearman) per hospital catchment area (HCA). In addition, we also report one distribution per lag time considered for estimating the correlation between DHRs and the considered temporal covariate. In practice, we investigated lag times ranging from 1 to 30 days. PM_2.5_ refers to particle matters ≤ 2.5 μm

A first visual comparison indicates an association between daily hospitalisation incidence and two temporal covariates: temperature and solar radiation (Additional file 1: Fig. S1). For each HCA, we then estimated the correlation between DHR and a temporal covariate while considering different lag times between the two measures (i.e. lag times ranging from 1 to 30 days; Fig. 5). The resulting density plots of correlation values highlight different trends in terms of temporal associations (Fig. 5): (i) DHR only becomes clearly positively correlated with the mobility index when considering a lag time of at least ~15 days, (ii) DHR is negatively correlated with daily temperature but not when considering important lag times (> 20 days), (iii) DHR is positively and negatively correlated with relative humidity and solar radiation, respectively, for almost all lag times considered. However, DHR tends to be negatively correlated with concentration of particles matter (≤ 2.5 μm) for lag times higher than 15–20 days.

## Discussion

Our analyses of the spatial heterogeneity in COVID-19 hospitalisation incidence mainly converge towards the relative importance of a particular spatial covariate: the ratio between the number of NH beds and the corresponding HCA population. In other words, HCAs with relatively higher proportions of NH beds tended to be associated with a higher hospitalisation incidence during the two epidemic waves of 2020. If we assume that the number of NH beds is a good proxy for the number of NH residents and that most hospitalised NH residents were admitted in the hospital of their HCA, this overall result further illustrates the strong impact of COVID-19 within NH communities on the Belgian hospitalisation burden. While it has been already established that nursing homes have to a large degree contributed to the Belgian COVID-19 death toll (> 57% of COVID-19 deaths in Belgium are nursing home residents), our results also illustrate their important contribution to the national hospitalisation burden, despite the fact that symptomatic NH residents have not all been hospitalised. According to the weekly nursing home reports released by Sciensano (https://covid-19.sciensano.be), a majority of COVID-19 deaths of NH residents (76% since the beginning of the crisis) indeed occurred in nursing homes rather than in hospitals.

Our study highlights that hospitalisation incidence was reasonably correlated between the two epidemic waves of 2020, but also that a BRT model based on spatial covariates and previous hospitalisation incidence can lead to good predictions of hospitalisation incidence (correlation between predicted and observed hospitalisation incidence > 0.85). The latter result should be interpreted with caution because we also show a tendency for an increase in incidence in HCAs that were relatively less impacted by the first epidemic wave. It is however difficult to interpret this tendency as it is based on the comparison between (i) hospitalisation incidence of the second wave and (ii) the difference in incidence between the second and first waves. These two measures are indeed by definition correlated because both are based on the incidence during the second wave.

Brussels-Capital Region is a particular case because it has several hospitals whose apparently small HCAs could not reflect the areas of origin of hospitalised people. Because of their relatively important capacities, especially its two major university hospitals, hospitals in Brussels also admitted patients from surrounding areas. For all these reasons, we also performed a new set of multivariate analyses without HCAs of Brussels in order to investigate to what extent they might have affected the outcome of our analyses (Additional file 4: Table S2). A comparison of the MLR and BRT results obtained with and without the HCAs of Brussels indicates no effect on our main conclusions.

The literature on hospitalisation incidence related to COVID-19 and spatial drivers is rather sparse. The few ecological studies describing the link between human and environmental drivers and COVID19, focus mostly on cases, mortality or case-fatality rates as outcomes. Also, to our knowledge, our study is the first indicating a significant association between the proportion of NH beds and hospitalisation incidence, even if some studies have shown a significant association between the proportion of older individuals and other COVID-19 indicators [17–19]. Other studies reported associations between various demographic and socio-economic factors [18–22] that are not confirmed in our analyses of hospitalisation incidence at the scale of Belgium. An association between urban areas and COVID-19 was also observed in Brazil, where the distance from the state capital (São Paulo) was negatively associated with COVID-19 prevalence [22].

In the second part of study, we explored the association between the evolution of hospitalisation incidence through time and several temporal covariates of interest. Our relatively descriptive analyses support the previously discussed links between viral circulation (here reflected by resulting hospitalisations) and weather conditions [23–25]: new hospitalisations were far lower in between the two epidemic waves of 2020, corresponding to the summer period associated with higher temperature and solar radiation. Although it is not straightforward to infer a causality due to several confounding factors that may come into play (e.g. behavioural), several studies have previously discussed such potential direct or indirect links between viral circulation and climatic factors such as temperature or solar radiation [23, 24, 26–28]. When considering a delay of at least ~ 15 days, our analyses also indicate that the evolution of new hospitalisations is associated with human mobility, which is not necessarily easy to interpret when looking at the overall epidemic period. Finally, our analyses find a negative and delayed association with concentrations of particulate matter, which is not in line with (nor necessarily contradicts) previous studies discussing the potential role of fine particulate matter in spreading the virus [29] or having a negative impact on COVID-19 symptoms [30].

The present study dealt with hospitalisation data only available at hospital level, and therefore used HCAs as units of analysis. This approach has some limitations. First, we assumed that patients were hospitalized in the closest hospital with a COVID-19 unit. However, other factors may influence hospital choices such as its size or reputation, especially in urban areas where the density of hospitals is higher. Second, the definition of HCAs is dependent on the friction surface used to calculate travel times. We used a global friction surface at a spatial resolution of approximately one by one km, which is based on default input data and modelling methods [14]. Such estimations may however vary with local contexts such as varying road speed limits or congestion. Another limitation relates to the way hospitalisations are recorded in Belgium. Some hospitals have several site locations, but COVID-19 patients were aggregated and only recorded at the main site. This may cause spatial discrepancies when a hospital receives COVID-19 patients on secondary sites. In addition, when hospitals were approaching saturation, patient transfers may have been organised resulting in hospitalisations recorded in more distant hospitals.

## Conclusion

Our study proposes a comprehensive analytical framework that can be applied to investigate dynamics and potential drivers of COVID-19 hospitalisation incidence at a regional or national scale. While our approach based on hospital catchment areas presents some limitations (discussed above), it allows performing spatially-explicit analyses of data aggregated at the hospital level. A better epidemiological understanding of the COVID-19 pandemic can serve as a guide in settling the most appropriate public health responses. Because most public health interventions aim at avoiding a saturation of hospitals, understanding the spatio-temporal dynamic as well as the potential drivers of COVID-19 hospitalisation incidence remains of crucial importance. In addition, we also illustrate that it is possible to get relevant predictions of hospitalisation incidence, which could be useful for a better planning of transfers among hospitals and thus avoiding potential saturation and local overflow.

## Supplementary Information

**Additional file 1: Figure S1.** Visual comparison between the evolution of daily new hospitalisations and the evolution of the temporal covariates considered in the present study. All temporal covariates were averaged over a distinct hospital catchment area (HCA), and each curve thus corresponds to a distinct HCA. All variables were also preliminary treated by a moving average of 7 days. The mobility index is based on mobile phone data (see the text for further detail); the temperature is reported in the Kelvin scale (K); the relative humidity is expressed as a percentage (ratio between vapor partial pressure and saturation vapor partial pressure; 100% meaning an air mass fully charged in humidity); and the solar radiation is reported in Joules per square metre (J/m^2^).

**Additional file 2: : Figure S2.** Principal component analysis (PCA) based on all spatial covariates as well as measures of hospitalisation incidence (HI). Specifically, we here included in the ACP HI values computed for the period corresponding to the first (01/03–31/05/2020) and the second (01/09–30/11/2020) epidemic waves. Each dot corresponds to a distinct hospital catchment area (HCA) and is displayed with an area proportional to the HI value computed for the entire epidemic period under consideration (01/03–30/11/20).

**Additional file 3: : Figure S3.** Response curves estimated for the boosted regression trees (BRT) model trained on measures of hospitalisation incidence (HI) computed for the entire epidemic period under consideration (01/03–30/11/2020).

**Additional file 4: Table S1.** Analyses of the potential predictors of spatial heterogeneity in hospitalisation incidence of nursing home (NH) residents. This table is equivalent to Table 1 and summarises the results of univariate linear regression (ULR), multivariate linear regression (MLR), and boosted regression trees (BRT) analyses performed to investigate the association between measures of hospitalisation incidence (HI) of NH residents and various spatial covariates associated with hospital catchment areas (HCAs). We report the following metrics: the coefficient of determination (R^2^) for the ULR analyses, the regression coefficient (β) for the MLR analyses, and the relative influence (RI) associated with each spatial covariate for the BRT analyses. In addition, we also report the overall R^2^ and Spearman correlation (“cor.”) for each distinct MLR and BRT analysis, respectively. (*) indicates if a given R^2^ or β is significant (p-value < 0.05).** Table S2. ** Analyses of the potential predictors of spatial heterogeneity in hospitalisation incidence, when excluding Brussels-Capital Region and a potential outlier area. This table is equivalent to Table 1 and summarises the results of univariate linear regression (ULR), multivariate linear regression (MLR), and boosted regression trees (BRT) analyses performed to investigate the association between measures of hospitalisation incidence (HI) and various spatial covariates associated with hospital catchment areas (HCAs). For these alternative analyses, we discarded the six HCAs of the Brussels-Capital Region, as well as a potential outlier HCA (marked with an asterisk in Fig. 4). We report the following metrics: the coefficient of determination (R^2^) for the ULR analyses, the regression coefficient (β) for the MLR analyses, and the relative influence (RI) associated with each spatial covariate for the BRT analyses. In addition, we also report the overall R^2^ and Spearman correlation (“cor.”) for each distinct MLR and BRT analysis, respectively. (*) indicates if a given R^2^ or β is significant (p-value < 0.05). **Table S3.** Comparison of the different boosted regression trees (BRT) models trained in the present study. Specifically, we here report the Spearman correlation between hospitalisation incidence predicted under various BRT models (one row = one specific BRT model) and observed hospitalisation incidence. As detailed in the text, the measure of hospitalisation incidence (HI) is computed as the cumulative number of new hospitalisations per 100,000 inhabitants for a given hospital catchment area (HCA) and a given time period. For each comparison between predicted and observed set of HI values, we report both the mean Spearman correlation value, as well as the minimum and maximum Spearman correlation values, all obtained while considering the ten BRT model replicates. (*) refers to the BRT model trained with HI values of the second epidemic wave (01/09-30/11/2020) but also including HI values of the first epidemic wave (01/03-31/05/2020) as a potential predictor in addition to all the other considered spatial predictors.

## Data Availability

R script and related files needed to run the analyses are all available at. https://github.com/sdellicour/covid19_spell. The GitHub repository also contains a tutorial for the delimitation of hospital catchment areas.

## Abbreviations

BRT: Boosted regression trees
DHR: Daily hospitalisation ratios
HCA: Hospital catchment area
HI: Hospitalisation incidence
MLR: Multivariate linear regression
NH: Nursing home
PCA: Principal component analysis
PM: Particulate matter
R^2^: Coefficient of determination
RI: Relative influence
ULR: Univariate linear regression
