# Supplementary figures and images for: Investigating the drivers of the spatio-temporal heterogeneity in COVID-19 hospital incidence—Belgium as a study case
Source: Int J Health Geogr. 2021 Jun 14;20:29. doi: 10.1186/s12942-021-00281-1 (PMC8200785; doi:10.1186/s12942-021-00281-1)

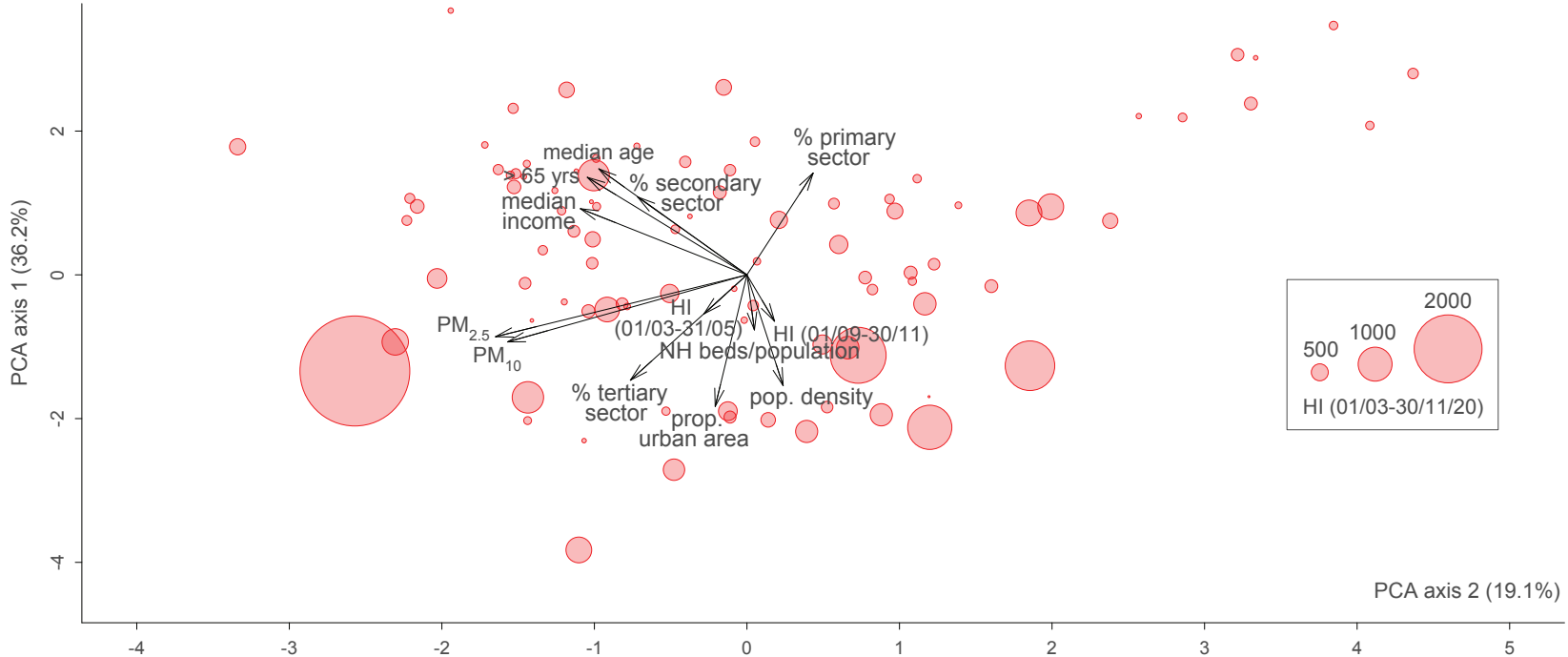

Supplement: Supplementary file 1 — Additional file 1: Figure S1. Visual comparison between the evolution of daily new hospitalisations and the evolution of the temporal covariates considered in the present study. All temporal covariates were averaged over a distinct hospital catchment area (HCA), and each curve thus corresponds to a distinct HCA. All variables were also preliminary treated by a moving average of 7 days. The mobility index is based on mobile phone data (see the text for further detail); the temperature is reported in the Kelvin scale (K); the relative humidity is expressed as a percentage (ratio between vapor partial pressure and saturation vapor partial pressure; 100% meaning an air mass fully charged in humidity); and the solar radiation is reported in Joules per square metre (J/m2). [file 12942_2021_281_MOESM1_ESM.pdf]

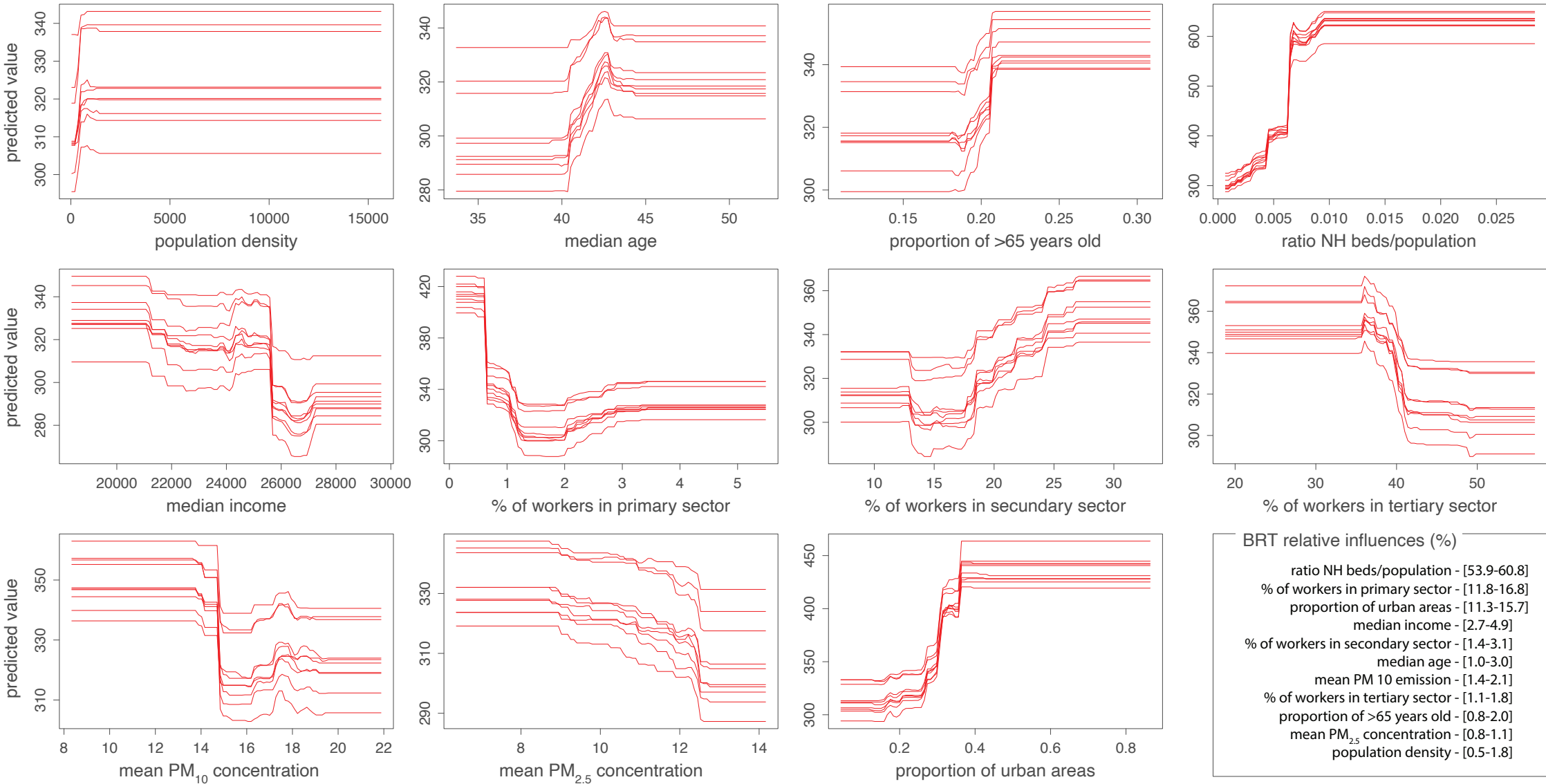

Supplement: Supplementary file 2 — Additional file 2: : Figure S2. Principal component analysis (PCA) based on all spatial covariates as well as measures of hospitalisation incidence (HI). Specifically, we here included in the ACP HI values computed for the period corresponding to the first (01/03–31/05/2020) and the second (01/09–30/11/2020) epidemic waves. Each dot corresponds to a distinct hospital catchment area (HCA) and is displayed with an area proportional to the HI value computed for the entire epidemic period under consideration (01/03–30/11/20). [file 12942_2021_281_MOESM2_ESM.pdf]

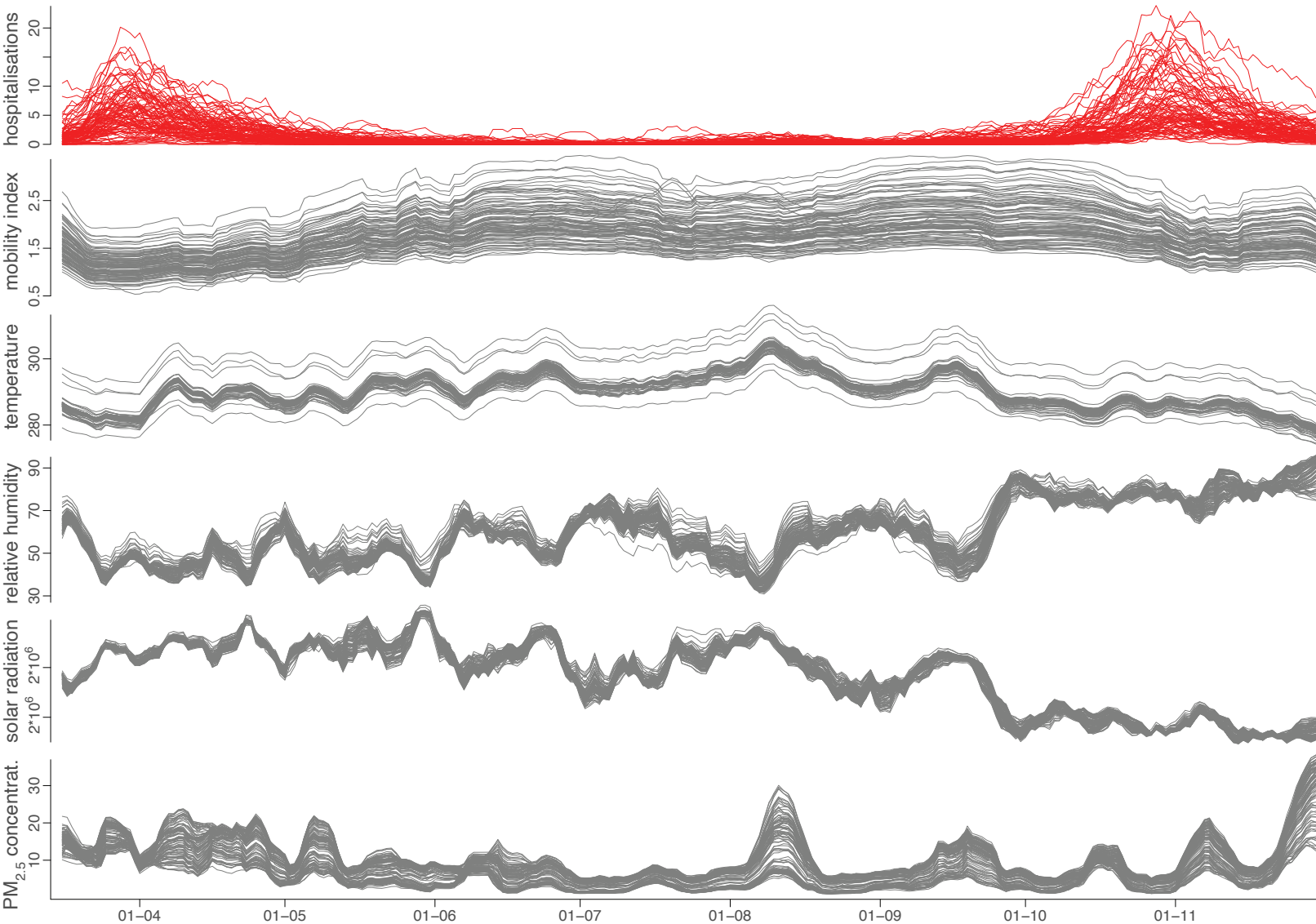

Supplement: Supplementary file 3 — Additional file 3: : Figure S3. Response curves estimated for the boosted regression trees (BRT) model trained on measures of hospitalisation incidence (HI) computed for the entire epidemic period under consideration (01/03–30/11/2020). [file 12942_2021_281_MOESM3_ESM.pdf]
