# Supplementary material for: Investigating the drivers of the spatio-temporal heterogeneity in COVID-19 hospital incidence—Belgium as a study case
Source: Int J Health Geogr. 2021 Jun 14;20:29. doi: 10.1186/s12942-021-00281-1 (PMC8200785; doi:10.1186/s12942-021-00281-1)
Supplement: Supplementary file 4 — Additional file 4: Table S1. Analyses of the potential predictors of spatial heterogeneity in hospitalisation incidence of nursing home (NH) residents. This table is equivalent to Table 1 and summarises the results of univariate linear regression (ULR), multivariate linear regression (MLR), and boosted regression trees (BRT) analyses performed to investigate the association between measures of hospitalisation incidence (HI) of NH residents and various spatial covariates associated with hospital catchment areas (HCAs). We report the following metrics: the coefficient of determination (R2) for the ULR analyses, the regression coefficient (β) for the MLR analyses, and the relative influence (RI) associated with each spatial covariate for the BRT analyses. In addition, we also report the overall R2 and Spearman correlation (“cor.”) for each distinct MLR and BRT analysis, respectively. (*) indicates if a given R2 or β is significant (p-value < 0.05). Table S2. Analyses of the potential predictors of spatial heterogeneity in hospitalisation incidence, when excluding Brussels-Capital Region and a potential outlier area. This table is equivalent to Table 1 and summarises the results of univariate linear regression (ULR), multivariate linear regression (MLR), and boosted regression trees (BRT) analyses performed to investigate the association between measures of hospitalisation incidence (HI) and various spatial covariates associated with hospital catchment areas (HCAs). For these alternative analyses, we discarded the six HCAs of the Brussels-Capital Region, as well as a potential outlier HCA (marked with an asterisk in Fig. 4). We report the following metrics: the coefficient of determination (R2) for the ULR analyses, the regression coefficient (β) for the MLR analyses, and the relative influence (RI) associated with each spatial covariate for the BRT analyses. In addition, we also report the overall R2 and Spearman correlation (“cor.”) for each distinct MLR and BRT analy [file 12942_2021_281_MOESM4_ESM.pdf]

**Table S1. Analyses of the potential predictors of spatial heterogeneity in hospitalisation incidence of nursing home (NH) residents.** This table is equivalent to Table 1 and summarises the results of univariate linear regression (ULR), multivariate linear regression (MLR), and boosted regression trees (BRT) analyses performed to investigate the association between measures of hospitalisation incidence (HI) of NH residents and various spatial covariates associated with hospital catchment areas (HCAs). We report the following metrics: the coefficient of determination ($R^2$) for the ULR analyses, the regression coefficient (β) for the MLR analyses, and the relative influence (RI) associated with each spatial covariate for the BRT analyses. In addition, we also report the overall $R^2$ and Spearman correlation ("cor.") for each distinct MLR and BRT analysis, respectively. (*) indicates if a given $R^2$ or β is significant (p-value < 0.05).

| | HI (01/03-31/05/2020) | | | HI (01/09-30/11/2020) | | | HI (01/03-30/11/2020) | | |
|---|---|---|---|---|---|---|---|---|---|
| | MLR $R^2$ = 0.18*, BRT cor. = 0.69 | | | MLR $R^2$ = 0.24*, BRT cor. = 0.76 | | | MLR $R^2$ = 0.23*, BRT cor. = 0.75 | | |
| **Spatial covariate** | **ULR $R^2$** | **MLR β** | **BRT RI** | **ULR $R^2$** | **MLR β** | **BRT RI** | **ULR $R^2$** | **MLR β** | **BRT RI** |
| population density | 0.02 | - | 2.0 % | 0.00 | - | 1.3 % | 0.00 | - | 0.9 % |
| median age | 0.03 | - | 2.4 % | 0.02 | - | 7.0 % | 0.03 | - | 5.3 % |
| prop. >65 years old | 0.03 | - | 11.5 % | 0.02 | - | 1.8 % | 0.02 | - | 1.5 % |
| ratio MR beds/population | 0.13* | 0.24* | 35.1 % | 0.22* | 0.43* | 52.6 % | 0.23* | 0.38* | 54.0 % |
| median income | 0.04 | - | 5.2 % | 0.06* | -0.09 | 6.5 % | -0.08 | - | 5.6 % |
| % in primary sector | 0.01 | - | 12.1 % | 0.04* | -0.01 | 13.8 % | 0.05* | 0.01 | 16.9 % |
| % in secondary sector | 0.01 | - | 15.8 % | 0.02 | - | 2.1 % | 0.02 | - | 2.1 % |
| % in tertiary sector | 0.02 | - | 3.1 % | 0.01 | - | 2.6 % | 0.02 | - | 2.1 % |
| $PM_{10}$ concentration | 0.01 | - | 5.5 % | 0.00 | - | 3.1 % | 0.00 | - | 2.6 % |
| $PM_{2.5}$ concentration | 0.01 | - | 1.2 % | 0.00 | - | 1.8 % | 0.00 | - | 1.3 % |
| prop. urban areas | 0.05* | 0.10 | 6.3 % | 0.05* | 0.08 | 7.5 % | 0.07* | 0.12 | 7.7 % |

**Table S2. Analyses of the potential predictors of spatial heterogeneity in hospitalisation incidence, when excluding Brussels-Capital Region and a potential outlier area.** This table is equivalent to Table 1 and summarises the results of univariate linear regression (ULR), multivariate linear regression (MLR), and boosted regression trees (BRT) analyses performed to investigate the association between measures of hospitalisation incidence (HI) and various spatial covariates associated with hospital catchment areas (HCAs). For these alternative analyses, we discarded the six HCAs of the Brussels-Capital Region, as well as a potential outlier HCA (marked with an asterisk in Figure 4). We report the following metrics: the coefficient of determination ($R^2$) for the ULR analyses, the regression coefficient (β) for the MLR analyses, and the relative influence (RI) associated with each spatial covariate for the BRT analyses. In addition, we also report the overall $R^2$ and Spearman correlation ("cor.") for each distinct MLR and BRT analysis, respectively. (*) indicates if a given $R^2$ or β is significant (p-value < 0.05).

| | HI (01/03-31/05/2020) | | | HI (01/09-30/11/2020) | | | HI (01/03-30/11/2020) | | |
|---|---|---|---|---|---|---|---|---|---|
| | MLR $R^2$ = 0.18*, BRT cor. = 0.69 | | | MLR $R^2$ = 0.24*, BRT cor. = 0.76 | | | MLR $R^2$ = 0.23*, BRT cor. = 0.75 | | |
| **Spatial covariate** | **ULR $R^2$** | **MLR β** | **BRT RI** | **ULR $R^2$** | **MLR β** | **BRT RI** | **ULR $R^2$** | **MLR β** | **BRT RI** |
| population density | 0.06* | -0.19 | 1.6 % | 0.02 | - | 0.9 % | 0.05* | -0.22 | 1.1 % |
| median age | 0.01 | - | 6.2 % | 0.05* | 0.03 | 8.7 % | 0.04 | - | 9.0 % |
| prop. >65 years old | 0.00 | - | 8.0 % | 0.02 | - | 2.4 % | 0.01 | - | 2.3 % |
| ratio MR beds/population | 0.31* | 0.19* | 33.9 % | 0.25* | 0.43* | 41.0 % | 0.32* | 0.27* | 45.8 % |
| median income | 0.01 | - | 4.4 % | 0.14* | -0.11 | 8.6 % | 0.09* | -0.01 | 4.8 % |
| % in primary sector | 0.04 | - | 11.9 % | 0.03 | - | 9.3 % | 0.04 | - | 8.4 % |
| % in secondary sector | 0.00 | - | 2.7 % | 0.00 | - | 2.5 % | 0.00 | - | 2.3 % |
| % in tertiary sector | 0.02 | - | 4.2 % | 0.00 | - | 8.6 % | 0.00 | - | 4.1 % |
| $PM_{10}$ concentration | 0.02 | - | 1.5 % | 0.00 | - | 4.2 % | 0.00 | - | 2.4 % |
| $PM_{2.5}$ concentration | 0.00 | - | 1.1 % | 0.02 | - | 4.6 % | 0.00 | - | 2.0 % |
| prop. urban areas | 0.08* | 0.22* | 24.6 % | 0.04 | - | 9.2 % | 0.07* | 0.27* | 17.8 % |

**Table S3. Comparison of the different boosted regression trees (BRT) models trained in the present study.** Specifically, we here report the Spearman correlation between hospitalisation incidence predicted under various BRT models (one row = one specific BRT model) and observed hospitalisation incidence. As detailed in the text, the measure of hospitalisation incidence (HI) is computed as the cumulative number of new hospitalisations per 100,000 inhabitants for a given hospital catchment area (HCA) and a given time period. For each comparison between predicted and observed set of HI values, we report both the mean Spearman correlation value, as well as the minimum and maximum Spearman correlation values, all obtained while considering the ten BRT model replicates. (*) refers to the BRT model trained with HI values of the second epidemic wave (01/09-30/11/2020) but also including HI values of the first epidemic wave (01/03-31/05/2020) as a potential predictor in addition to all the other considered spatial predictors.

| Predictions obtained with a BRT model trained with | HI observations 01/03-31/05/20 | HI observations 01/09-30/11/20 | HI observations 01/03-30/11/20 |
|---|---|---|---|
| **HI - 01/03-31/05/20** | 0.69 [0.65-0.72] | 0.49 [0.48-0.49] | 0.68 [0.65-0.69] |
| **HI - 01/09-30/11/20** | 0.42 [0.41-0.43] | 0.76 [0.75-0.77] | 0.68 [0.67-0.69] |
| **HI - 01/09-30/11/20*** | 0.68 [0.67-0.68] | 0.86 [0.86-0.87] | 0.89 [0.89-0.89] |
| **HI - 01/09-30/11/20** | 0.60 [0.57-0.62] | 0.69 [0.67-0.71] | 0.75 [0.73-0.77] |
